# Circum‐Setouchi conference: Transboundary support for residents in small‐scale programs

**DOI:** 10.1002/jgf2.407

**Published:** 2020-11-20

**Authors:** Junki Mizumoto

**Affiliations:** ^1^ Department of Medical Education Studies International Research Center for Medical Education Graduate School of Medicine The University of Tokyo Tokyo Japan

To The Editor:

Most resident physicians in community hospitals in Japan tend to feel vaguely anxious about their achievement because they lack opportunities to attend study groups and have irregular supervision owing to a shortage of doctors compared with residents in large‐scale programs.^1^

The Chugoku and Shikoku branch of the Japan Federation of Democratic Medical Institutions has 10 postgraduate education hospitals across eight prefectures. Each hospital is relatively small‐scale and has up to four residents in each grade. To help residents in the education hospitals improve their basic practical skills, share experiences, and interact with each other, we have held monthly resident meetings (the “Circum‐Setouchi Conference”) since 2002. The word “uchi” means inland in Japanese and refers to the Seto Inland Sea, which is surrounded by the eight prefectures.

Up to 2019, this conference had been held on a face‐to‐face basis. Approximately 7‐15 participants attended each conference. Participant reports indicated the following. Almost all participants found the conferences satisfying and interesting. Many participants placed greater value on interaction with residents from other hospitals than on knowledge acquisition, because they felt the interactions ameliorated the isolation they felt during the small‐scale residency program. Participants often learned about the curricula of other hospitals and then reflected on their own training. Additionally, the intimate and blame‐free atmosphere encouraged active learning.

From February 2020, the conference has been held online because of SARS‐CoV‐2. Participants report that the online conference has several advantages. First, the participation barrier of long‐distance travel is removed. In fact, the number of participants has increased to over 20. Second, participants feel more relaxed “attending” from their own rooms. Third, and most surprisingly, most residents report that typing in a chat window is more comfortable than verbally expressing their opinions. There are two probable reasons why they feel comfortable in chat discussion. One is that, in online conferences with most participants' videos off for reducing the amount of data traffic, knowing what the other members are doing is often difficult. The other is that, according to some residents' reports, they are used to typing short messages using social networking services and other electronic media. Using chat in online case‐based teaching conferences may develop learners' more positive attitude to their own achievement.^2^

The conference style has therefore changed. A facilitator poses appropriate questions depending on the case. Residents post their answers in a chat window. The facilitator reads the responses aloud then adds comments to each one, as a radio host would read letters from listeners. This style may have some potential advantages. Firstly, the facilitator's comments encourage participants to express their own opinions. Secondly, the length of discussion time is saved. Thirdly, the original roles of this conference are maintained because participants can freely use chat window to communicate with each other.

In summary, this multi‐institutional joint teaching conference provides residents with opportunities to communicate with each other and reflect on their own training. Online conferences in the current SARS‐CoV‐2 environment may have several advantages, including activated discussions emerging from chat responses.

## CONFLICT OF INTEREST

The authors have stated explicitly that there are no conflicts of interest in connection with this article.
